# Multiscale Simulation of Nanowear-Resistant Coatings

**DOI:** 10.3390/ma18143334

**Published:** 2025-07-16

**Authors:** Xiaoming Liu, Kun Gao, Peng Chen, Lijun Yin, Jing Yang

**Affiliations:** 1 Inner Mongolia Power (Group) Co., Ltd., Inner Mongolia Power Research Institute Branch, Hohhot 010020, China; 2 School of Chemical Engineering and Technology, Sun Yat-sen University, Zhuhai 519082, China; gaok8@mail2.sysu.edu.cn

**Keywords:** wear-resistant coating, atomic scale, macroscale, multiscale simulation

## Abstract

Nanowear-resistant coatings are critical for extending the service life of mechanical components, yet their performance optimization remains challenging due to the complex interplay between atomic-scale defects and macroscopic wear behavior. While experimental characterization struggles to resolve transient interfacial phenomena, multiscale simulations, integrating ab initio calculations, molecular dynamics, and continuum mechanics, have emerged as a powerful tool to decode structure–property relationships. This review systematically compares mainstream computational methods and analyzes their coupling strategies. Through case studies on metal alloy nanocoatings, we demonstrate how machine learning-accelerated simulations enable the targeted design of layered architectures with 30% improved wear resistance. Finally, we propose a protocol combining high-throughput simulation and topology optimization to guide future coating development.

## 1. Introduction

Metal wear is a prevalent issue in critical industries such as the chemical sector, metallurgy, and electric power generation, impacting various equipment components like pumps, turbines, and reactors. Due to prolonged operation under challenging conditions, which can be characterized by high temperatures, wear, erosion, and thermal shocks, metal equipment often necessitates costly repairs or replacements, resulting in significant economic losses [1,2,3].

Recent advancements in nanotechnology have opened new paths in surface engineering, particularly through the integration of nanomaterials with coating technologies [4,5]. This combination not only catalyzes future enhancements in coating methods but also facilitates the expansion of nanomaterials that possess exceptional properties for diverse applications. For instance, low-dimensional and functionalized nanostructured coatings exhibit specific attributes, including mechanical strength, thermal resistance, acoustic insulation, and electrical conductivity, which can substantially improve material characteristics such as surface hardness, adhesion strength, high-temperature corrosion resistance, and tribological performance [6,7,8]. Hence, nanocoatings have emerged as a focal point of international research, directing attention toward the design and synthesis of innovative nanocoating materials.

In the field of nanocoating design and synthesis, accurately describing the atomic and chemical combinations that form solid surfaces is critical. Each nanostructural design contains varying interfaces that impart distinct optical, electrical, magnetic, and mechanical properties, differing considerably from those found in bulk materials. A central task in this area is to clarify the physical nature of the phenomena at these interfaces, as many challenges in nanosurface engineering cannot be captured through experiments alone. By analyzing atomic-scale dynamics, researchers can gain insights into the microscopic mechanisms [9,10,11] necessary for the development of innovative materials. This involves careful design at solid–solid [12] and solid–liquid [13] interfaces, focusing on managing factors like friction, wear, oxidation, and corrosion, as well as enhancing lubrication and dynamic contact processes. The complexity of these interactions necessitates the application of advanced theoretical methodologies from nanophysics. As computer simulations are not bound by the practical limitations of material testing, they play a vital role in examining the transition from elastic to plastic deformation at the atomic level, thereby facilitating significant advances in nanocoating research.

The existing continuum model based on macroscale perspectives cannot effectively capture the microscopic deformation mechanisms, including dislocation nucleation in crystals, dislocation movement, slip surface dynamics, and potential chemical reactions on coating surfaces [14,15,16]. This inadequacy poses challenges in accurately characterizing the microscopic properties of materials. Atomic-scale simulation methodologies, which draw upon quantum mechanics, molecular dynamics, and Monte Carlo methods, offer potent avenues for studying nanocoatings. However, the relatively large spatial and temporal scales often used in these simulations can limit their ability to account for critical microscale phenomena, largely due to computational limitations. Consequently, the emergence of multiscale simulation methods that integrate both atomistic and continuum approaches is crucial [17]. This article reviews theoretical calculations, multiscale simulation strategies, and their applications within the realm of nanocoatings, underscoring their significance in advancing materials science research.

## 2. Theoretical Models and Calculation Methods at Different Levels of Nanomaterials

**Figure 1:**
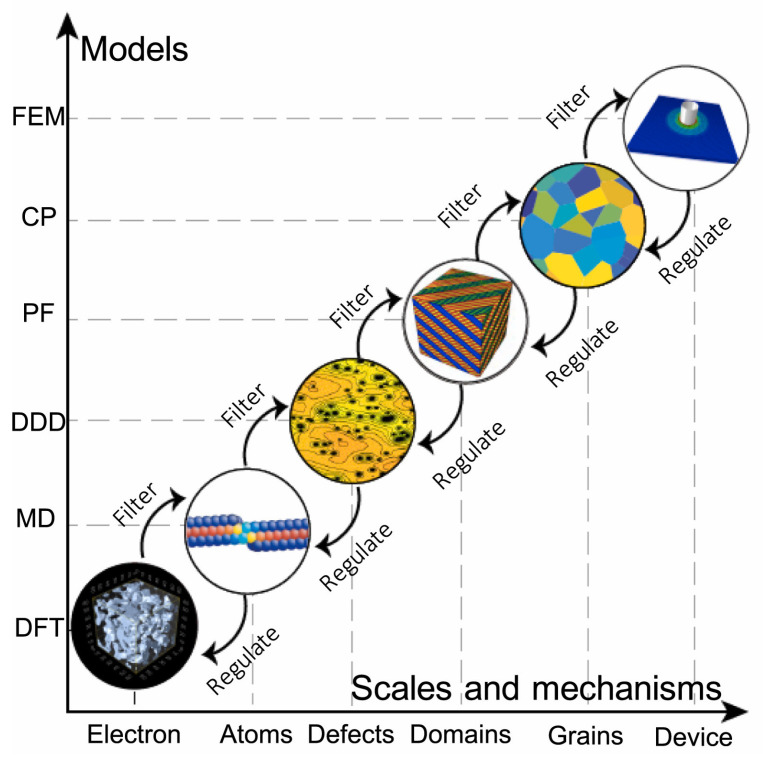
Multiscale modeling of materials is a ‘divide-and-conquer’ approach to describe the complexity of material behavior. Reprinted with permission from Ref. [24]. Copyright 2021, Elsevier Ltd.

The classical analysis and calculation models in material mechanics are based on a continuum medium, relying on macroscopic constitutive models independent of the size of the model. Simulation analysis typically involves selecting a constitutive model that accurately captures the macroscopic behavior of materials while managing boundary conditions effectively. However, at the nanoscale, nanomaterials experience significant changes in hardness, strength, and ductility [18]. Numerous experimental and numerical simulations suggest that the scale effect alters the deformation mechanisms of materials, closely related to their internal structures [19,20]. To address this cross-scale challenge, it is imperative to establish a new paradigm that integrates classical continuum mechanics, microscopic computational techniques, and concurrent multiscale methodologies. This necessitates advancing continuum theories incorporating micromechanical principles while simultaneously enhancing continuum constitutive models through atomistic parameters derived from quantum mechanical calculations. Figure 1 illustrates the concrete implementation of this paradigm via a “divide-and-conquer” strategy [21,22,23]. Complex material behavior is first deconstructed into ordered multiscale hierarchies. Dedicated theoretical tools are then independently applied at each scale to identify governing mechanisms. Finally, cross-scale knowledge integration is achieved through bidirectional information transfer between adjacent scales. Building upon this methodological framework, this section will systematically elaborate on the design principles for nanomaterials and the development of corresponding computational approaches.

### 2.1. First-Principles Simulations

Many fundamental physical properties of materials stem from their electronic structure, which can be determined using first-principles calculation methods [25,26,27]. In recent years, first principles have played an important role in theoretical predictions of new materials. As nanostructured materials exhibit distinct mechanical behaviors governed by quantum mechanics rather than classical Newtonian mechanics, their electronic structures can also be accurately computed through first-principles approaches. Furthermore, these methods can calculate the unit cell size of crystals with an acceptable error margin of a few percent, along with other geometric structural aspects like the positioning of impurities, dislocations, defect structures, grain interfaces, and surfaces.

**Figure 2:**
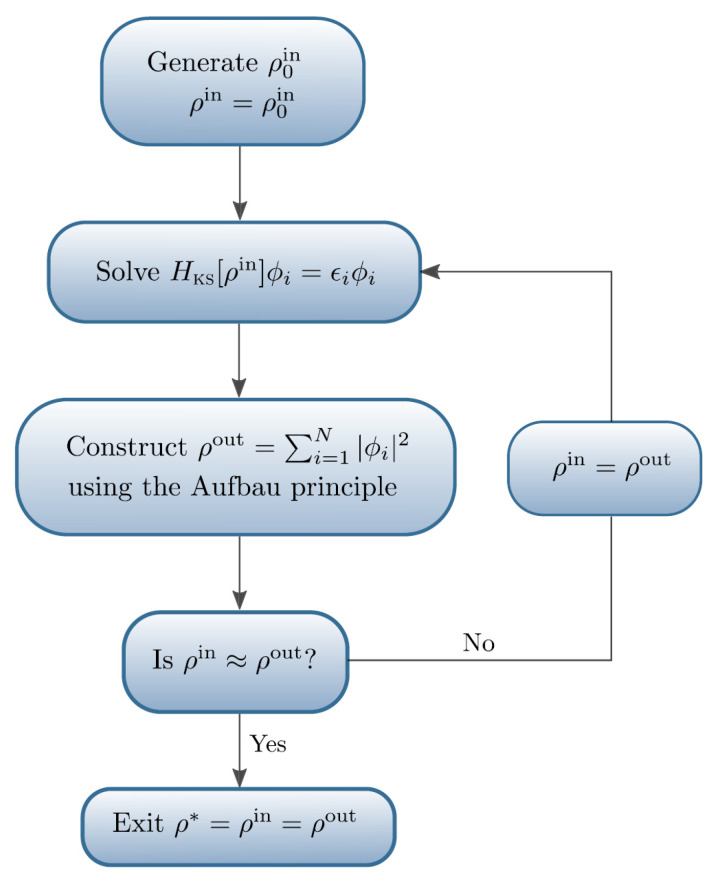
A flowchart detailing an example algorithm for achieving self-consistency using fixed-point (or Roothaan) iterations. Reprinted from Ref. [31].

Density functional theory (DFT) is a fundamental method for first-principles calculations, essential for studying the ground state of multi-particle systems as per the Hohenberg–Kohn theorem [28]. This theory effectively converts the intricate multi-body problem of electron–nucleus interactions into a series of coupled single-particle equations known as the Kohn–Sham (KS) equations. These equations are solved through a process of self-consistent calculations, detailed in Figure 2, which illustrates the calculation flow. DFT enables parameter-free assessments of crucial ground-state physical observables, such as the elastic modulus, the vibration frequency, defects, and surface properties. It plays a vital role in the development of nanocoatings, enhancing their performance and functional properties by enabling precise modeling at the nanoscale. For example, Wang et al. [29] demonstrated for the first time the successful growth of high-quality monolayer NbSe_2_ with superlubricity and superhigh wear resistance in an ambient atmosphere via chemical vapor deposition (CVD). DFT calculations revealed that the adhesion force and degree of charge transfer between the NbSe_2_ monolayer and the substrate were greater than those between the topmost layer and multilayer NbSe_2_ underlayers. Peng et al. [30] achieved an extensive and controllable reduction in friction in atomically thin graphene via pre-sliding under high stress. Compared with pristine graphene, the coefficient of friction was reduced by six-fold. DFT calculations attribute this friction reduction to a decreased sliding barrier and an increased contact stiffness. These effects result from the enhanced adhesion strength between graphene and the substrate due to interfacial charge transfer.

#### 2.1.1. Calculation of Mechanical Properties

Elastic modulus: The stress experienced by the crystal under the action of an external force and the resulting deformation can be characterized by the stress tensor, strain tensor, and elastic stiffness coefficients (elastic constants) as follows:

(1) $$\sigma_{\mathrm{ij}}\;={\;C}_{\mathrm{ijkl}}\varepsilon_{\mathrm{kl}}$$

Elastic coefficients are calculated through the framework of continuous elastic theory, which applies a uniform strain derived from the system’s equilibrium state. This allows for the calculation of the stress–strain relationship and the subsequent derivation of the elastic modulus. For three-dimensional materials, the elastic stiffness constant matrix consists of 6 × 6 components [32], while the count of independent elastic constants depends solely on the crystal system and is independent of the specific symmetry types. In the case of cubic crystal systems, for example, only three independent elastic constants exist: C_11_, C_12_, and C_44_. The elastic modulus can be obtained using the Voigt–Reuss–Hill [32,33] approximation applied to these constants.

Ideal strength [34] and hardness [35]: As materials and structures scale down to the nanoscale, their defects significantly diverge from those typical of bulk materials, necessitating a re-evaluation of ideal strength and hardness parameters. The low-temperature strength of materials can be enhanced through methods such as surface annealing, the optimization of operational parameters, and the exploration of alternative synthesis or processing routes. In line with this trend, the defect density of bulk materials approaches zero as they attain their ideal strength. Utilizing first-principles calculations, researchers can determine the maximum stress value that a uniformly deformed crystal unit cell can withstand, which defines its ideal strength. Hardness, a key physical property, indicates a material’s capacity to resist both elastic and plastic deformation. This property is intricately linked to the material’s microstructural and macrostructural characteristics. Notably, Chen and his colleagues [36] developed a hardness formula that quantifies these relationships, which is shown below:

(2) $$H_{V}\;=\;CK^{m}G^{n};K=G/B$$

where H_v_, G, and B are the hardness (GPa), shear modulus (GPa), and bulk modulus (GPa), respectively. C, m, and n are empirical coefficients, and *K* is the ratio of Pugh’s modulus *G*/*B*. This formula demonstrates outstanding performance in predicting the hardness of superhard materials (e.g., diamond and BC2N), transition metal carbides and nitrides (e.g., TiN and WC), and bulk metallic glasses, with prediction errors typically below 10% for the 39 crystalline compounds evaluated, as demonstrated in this paper.

#### 2.1.2. Calculation of Chemical Properties

Corrosion resistance in metals [37] refers to the ability of these materials to withstand corrosive damage from their surroundings. This property is influenced by various factors, including the material’s composition, chemical characteristics, and microstructure. Typically, metals and alloys that display high corrosion resistance fall into one of three categories: (1) those with high thermodynamic stability, such as Pt, Au, Ag, Cu, and certain alloy systems like Au-Cu and Ni-Cu-Cr; (2) metals that easily undergo passivation, such as Ti, Zr, Ta, Nb, Cr, and Al, including chromium-containing stainless steel; and (3) metals that form well-protected, insoluble corrosion product membranes. However, much of the current knowledge on corrosion-resistant materials is still based on macroscopic and empirical observations, lacking insight from micro-, nano-, molecular, and atomic levels. DFT presents an effective tool for examining the corrosion behavior of materials at these smaller scales [38,39,40,41], enabling researchers to accurately compute and analyze the fundamental mechanisms of metal corrosion at the atomic level [42].

The work function [43] is a critical parameter influencing the corrosion behavior of materials and is closely linked to corrosion potential. It refers to the minimum energy needed to remove electrons from a solid surface to a point in a vacuum, effectively indicating the ability of a substance to bind electrons. A higher work function signifies that it is more challenging for the material to lose electrons. According to the latter definition, the work function can be expressed as Equation (3), representing the energy required to move electrons from the Fermi level to a vacuum. It represents the ability of a substance to bind electrons. The greater the work function, the harder it is for electrons to leave, and accordingly, the harder it is to be corroded.

(3) $$W={\;E}_{\mathrm{vacuum}}-{\;E}_{\mathrm{fermi}}$$

Yan et al. [44] made significant advancements in abradable seal coating corrosion with their CuAl-Ni/C sealing coating. By partially substituting Al with Cu in the Al-BN coating, they achieved a coating known for excellent abrasion resistance, erosion resistance, and high-temperature oxidation resistance. The intermediate phase present in the coating notably enhanced its resistance to corrosion at room temperature. Interestingly, it was observed that, during early-stage corrosion, copper actively dissolves at a higher rate than aluminum, a trend that contradicts the conventional understanding of their respective activities. The researchers employed first principles to compute the work functions of various crystal faces and their chlorine ion adsorption energies. Notably, Cu_4_Al(110) exhibited a low work function along with the most negative adsorption energy, highlighting that the higher dissolution rate of Cu compared with Al is primarily due to the preferential corrosion of the Cu_4_Al intermediate phase.

### 2.2. Molecular Dynamics Simulation

Molecular dynamics (MD) operates on the principles of Newtonian mechanics and is recognized as an efficacious computational simulation method for probing the physical behavior of materials along with their internal microscopic mechanisms at the atomic scale. Primarily, this technique relies on interatomic potentials, including but not limited to the Tersoff potential, to calculate the forces on individual atoms. By integrating Newton’s equations of motion, it provides data on atomic velocities, coordinates, and trajectories, enabling a comprehensive analysis of the physical and mechanical behaviors of materials through statistical mechanics. Molecular dynamics adeptly tracks real-time atomic motion, investigates microstructures, and identifies defects such as dislocations and voids, thus revealing the intricate microscopic details that correspond to the apparent physical and mechanical responses observed in materials [45].

#### 2.2.1. Typical Interatomic Potential

The central challenge in molecular dynamics simulation is the accurate determination of interatomic potentials. The direct solution of the Schrödinger equation for complex systems remains highly demanding. Consequently, potentials are typically derived through experimental fitting or semiempirical methods to obtain the system energy. Given that most nanocoatings involve metallic materials, the embedded atom method (EAM) potential has become one of the most widely used descriptions for metals. Proposed by Daw and Baskes [46], the EAM model treats each atom in the system as an impurity embedded within a host lattice formed by all other atoms. Within this framework, the total potential energy comprises two key contributions: a pairwise potential energy and an embedding energy. The embedding energy term is a function of the local electron density, thereby capturing the interaction between the embedded atom and its host environment, representing the essential many-body effects. The model assumes that the host electron density is a linear superposition of the electron densities contributed by the constituent atoms. These atomic electron densities are approximated by the spherically averaged densities of their valence electrons, particularly s and d electrons. The EAM potential describes the total potential energy of the system as follows:

(4) $$E_{T}\;=\;\frac{1}{2}\sum_{i,j(i\neq j)}V\left(r_{\mathrm{ij}}\right)\;+\sum_{i}F_{i}\left(\rho_{i}\right)$$

In Equation (4),

(5) $$\rho_{i}=\sum_{i,j(i\neq j)}f(r_{\mathrm{ij}})$$

In the formula, V(r) represents the interaction potential energy of the two bodies, $F_{i}\left(\rho_{i}\right)$ represents the embedding energy, f(r) represents the spherical symmetry of the distribution function, $\rho_{i}$ represents the local electron density at the location of atom i, and $r_{ij}$ represents the distance between two atoms, i and j.

The EAM potential effectively captures the electronic structure and chemical bonding characteristics of metals. It is particularly well-suited for modeling interatomic interactions and defect behaviors within metallic crystals. Furthermore, the EAM framework proves valuable for investigating phenomena such as high-temperature melting, solid–liquid phase transitions, and the properties of metal surfaces and interfaces. While the EAM model does not explicitly account for factors like electronic correlation and non-local effects, limiting its ability to describe complex electronic structures or subtle electronic behaviors, it remains the most widely employed potential for simulating metallic materials.

#### 2.2.2. Simulation of Materials Friction Properties

Molecular dynamics (MD) simulations have proven highly effective in elucidating the fundamental friction and wear mechanisms of metals. Key simulation studies [47,48,49,50] attribute material deformation during friction to elastic deformation, atomic-scale amorphization, and dislocation slip within the contact region. Consequently, MD provides a powerful tool for analyzing friction and wear processes at the atomic level. For instance, Lou et al. [51] employed MD simulations to investigate the tribological behavior and microscopic wear mechanisms of WC-Co cemented carbide. By systematically varying model parameters such as the WC grain size, applied load, and sliding velocity, their study offered deep insights into the root causes of wear failure in WC-Co materials.

**Figure 3:**
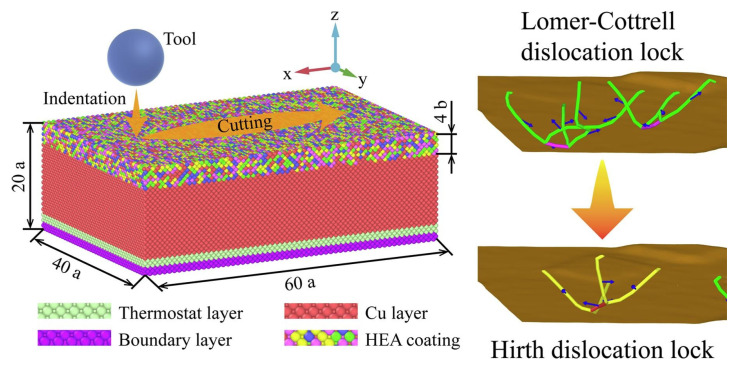
High-entropy alloy friction simulation modeling. Reprinted with permission from Ref. [52]. Copyright 2021, Elsevier B.V.

Similarly, Li et al. [52] discovered through MD (molecular dynamics) research that the friction coefficient of FeNiCrCoCu high-entropy alloy coatings decreased with increasing cutting speed (Figure 3). This phenomenon is attributed to the competing effects of thermal softening and strain rate hardening at high speeds. Additionally, increasing the cutting depth led to more atoms coming into contact with the abrasive grain, significantly increasing the friction coefficient. Zihan Li [53] applied MD simulations to examine the tribological properties of a tungsten disulfide (WS_2_)-coated copper system, aiming to understand its nanoscale wear mechanism. Their approach involved comparing the mechanical responses of bare copper and WS_2_-coated copper during indentation. Furthermore, they analyzed the influence of the nanoindentation depth and the anisotropic orientation of the WS_2_ coating on the friction behavior. These examples demonstrate that MD simulations enable the precise measurement of diverse nanoscale mechanical properties beyond friction, including load-displacement curves, the elastic modulus, hardness, and fracture toughness.

#### 2.2.3. Material Fracture Deformation Simulation

Fracture is a complex, multiscale phenomenon. While macroscopic fracture behavior has been extensively researched, a fundamental understanding of microscopic fracture mechanisms and the associated evolution of the stress field during fracture remains less developed. To address this gap, Wu et al. [54] employed molecular dynamics (MD) simulations to investigate the fracture behavior and stress distribution characteristics of single-crystal nickel containing distinct initial defects, namely, sharp cracks, blunt cracks, and nanoscale voids. The simulation results demonstrate that these initial defect geometries significantly influence the governing fracture mechanisms, fracture strength, and fracture toughness of the material.

Liu et al. [55] employed quasi-3D molecular dynamics (MD) simulations to investigate crack propagation in gradient nanocrystalline copper. Their model featured a centrally located initial crack and underwent uniaxial tensile deformation. By systematically comparing crack propagation rates and contrasting the results with uniformly grained specimens of varying grain sizes, the study elucidated the significant influence of grain size gradients on crack growth behavior, revealing a critical grain size threshold that governs the transition in propagation mechanisms. To unravel the tensile fracture mechanisms at the crystalline–amorphous interface in Cu-Zr-based metallic glass composites, Zhang et al. [56] conducted MD simulations of the interfacial deformation behavior. Their findings indicated that grain boundaries (GBs) within the crystalline phase are susceptible sites for local temperature rises and stress concentration, triggering amorphization. Concurrently, a reversible martensitic transformation occurs within the crystalline phase. While dislocation accumulation at the interface induces work hardening in the crystalline phase, the concomitant GB amorphization reduces the work-hardening rate. Crucially, when the applied stress exceeds the hardening capacity at the interface, fracture initiates preferentially at the crystalline phase grain boundaries along the bimaterial interface.

### 2.3. Monte Carlo Method

The Monte Carlo (MC) method, also known as stochastic simulation or statistical sampling, is a computational technique rooted in probability theory and mathematical statistics. It employs random sampling via computer simulations to estimate the statistical properties of a target system. The fundamental concept of the MC method involves representing the solution to a problem—be it in mathematics, physics, chemistry, or materials science—as a probability model or stochastic process. The desired solution is then equated to either the probability of a specific random event occurring or the expected value (mathematical expectation) of a relevant random variable.

Unlike molecular dynamics (MD), the Monte Carlo (MC) method avoids iteration-related challenges and exhibits virtual immunity to numerical instability. Its convergence is guaranteed as N→∞ (where N denotes the number of particles), though convergence to the correct solution depends critically on model validity. Notably, MC convergence rates are dimensionally independent, a significant advantage over grid-based methods. Further benefits include straightforward error estimation and a substantially lower computational expense compared with MD, requiring significantly less machine time. The method’s computational simplicity makes it equally suitable for deterministic problems and uniquely powerful for stochastic systems. For instance, Wang et al. [57] employed Monte Carlo (MC) simulation to evaluate the reliability of thermal barrier coatings (TBCs) in aero-engine combustors under high-temperature gas flow. The study defined coating failure as the thermally grown oxide (TGO) layer reaching a critical thickness of 10 μm. By accounting for uncertainties in the combustor wall temperature distribution and material property variations, they established a probabilistic failure model. The simulation results demonstrated a failure probability growth trend that was highly consistent with borescope inspection measurements, validating the method’s reliability and confirming the decisive influence of temperature and TGO growth on coating lifespan.

**Figure 4:**
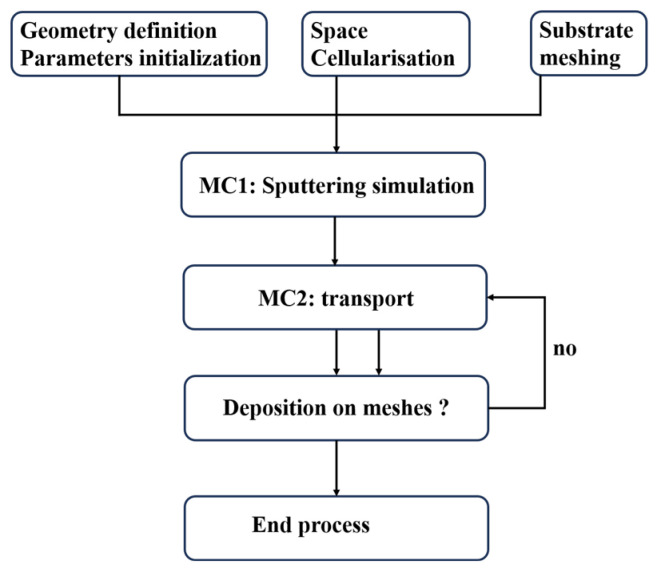
Monte Carlo algorithm for predicting film thickness deposited by physical vapor deposition (PVD). Reprinted with permission from Ref. [61]. Copyright 2019, Elsevier B.V.

Through extensive random sampling, the MC method computationally “replicates” the coating’s evolution pathway from initial nucleation to final microstructure formation. This approach predicts critical coating characteristics including surface morphology, porosity, grain size, and columnar grain structure. Serving as an effective tool for understanding coating formation mechanisms, optimizing process parameters, and predicting microstructure–property relationships, its application potential continues to expand. MC simulations effectively model material-specific random processes such as nanofilm growth (Figure 4), atomic diffusion, defect dynamics, phase transformations, and collision phenomena [58,59,60].

### 2.4. Finite Element Simulation

**Figure 5:**
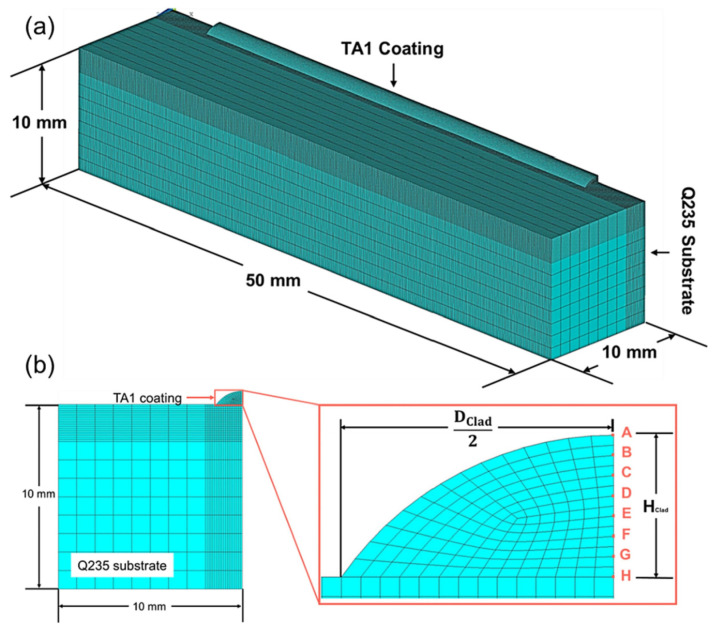
Meshed model of the substrate with a single track coating: (**a**) the overall view and (**b**) the front view, including the partially enlarged view of the cross-section of the coating. Reprinted with permission from Ref. [62]. Copyright 2022, Elsevier B.V.

The finite element method (FEM) is a well-established numerical technique that employs spatial discretization and piecewise polynomial interpolation to formulate unified governing equations for continuous media, enabling efficient computational solutions. For instance, Zuo-Jiang et al. [62] developed a novel 3D FEM framework (Figure 5) simulating laser cladding processes. This model simultaneously resolves thermal fields, computes solidification parameters at moving solid–liquid interfaces, and analyzes microstructural evolution. In another application, M. Rusinowicz and coworkers [63] investigated nanoindentation-induced fracture in Si_3_N_4_/AlSiCu/SiO_2_/Si multilayer systems. Their extended FEM (XFEM) implementation, incorporating cohesive zone modeling, captured three key phenomena: (1) plastic deformation within the AlSiCu layer; (2) crack initiation and propagation in the Si_3_N_4_ layer; and (3) interfacial delamination at the Si_3_N_4_/AlSiCu interface. Notably, characteristic “pop-in” events were observed in the load–displacement response. Sumit Bhattacharya et al. [64] developed a refined three-dimensional thermomechanical coupling model using the COMSOL 5.3a Multiphysics platform, successfully revealing stress evolution patterns in ZrN coatings on U-Mo fuel particle surfaces. The model specifically compared two typical scenarios, namely, the presence and absence of an Al_2_O_3_ interlayer. The results indicated that the absence of an interlayer generated substantial stress gradients up to 350 MPa in the ZrN coatings due to thermal expansion coefficient mismatch. This stress concentration phenomenon correlates closely with SEM-observed semi-ellipsoidal UO_2_ oxide distributions. Leveraging finite element simulations, the researchers analyzed how coating thickness, material combinations, and interface structures affect mechanical performance, thereby informing the design of gradient coatings or multilayer architectures. This methodology further enables the simulation of the effects of process parameters (e.g., spraying and deposition) on residual stresses and adhesion strength, uncovering the root causes of coating delamination and cracking while significantly reducing experimental trial-and-error costs.

### 2.5. Summary

The evolution of multiscale simulation methods for nanowear-resistant coatings represents an attempt by computational materials science to break through the inherent dilemma of balancing “accuracy, efficiency, and scale.” Density functional theory (DFT), based on quantum mechanics principles, can precisely reveal the intrinsic properties at the electronic scale. However, its prohibitively high computational cost severely limits its practical application in engineering contexts. Molecular dynamics (MD) simulations offer atomic-level resolution, dynamically capturing key failure mechanisms such as dislocation slip and crack nucleation. Nevertheless, their limitation to nanosecond time scales renders them incapable of addressing engineering lifespan problems. The Monte Carlo (MC) method efficiently handles stochastic processes like deposition nucleation and grain boundary evolution through probabilistic sampling, but it sacrifices physical authenticity by neglecting real dynamic mechanisms. While finite element analysis (FEA) constructs a comprehensive picture of the macroscopic stress field, it relies on empirical constitutive models that lack causal links to underlying atomic mechanisms, effectively operating as a “black box”. Table 1 also details comparative analyses. These methods, while successful within their specific spatiotemporal domains, exist in a stand-alone yet fragmented state. This fragmentation starkly exposes the core challenge in material simulation: When the ultimate performance of a coating arises from cross-scale interactions, simulation methods confined to any single scale can only provide partial understanding, failing to grasp the complex behavior of the entire system.

## 3. Multiscale Simulation

The fundamental disconnect between atomistic and continuum approaches necessitates multiscale simulation for comprehensively studying materials. Emerging as a distinct discipline in recent decades, multiscale modeling builds upon earlier efforts to connect macroscopic behavior with atomic interactions. Its primary strategies are hybrid (parallel) methods and hierarchical methods.

### 3.1. Hybrid Modeling

Hybrid multiscale methods resolve distinct material regions using appropriate simulation techniques. Addressing a different scale-bridging challenge to address different scale-bridging challenges, the quasicontinuum (QC) method [75,76] was developed. In the QC method, the core of data transfer between the atomistic and continuum scales relies on the synergistic mechanism of adaptive finite element meshing and representative lattices. Within the continuum regions (coarse meshes), the Cauchy–Born rule is employed: the local deformation gradient tensor F uniformly maps lattice atom positions, directly converting the atomistic potential energy into continuum energy density and stress. Conversely, in non-uniform regions, such as defect cores or interfaces (fine meshes), the non-local mode is activated: the displacement of each atom is precisely interpolated via finite element shape functions, and atomistic-level energy and force fields are computed directly based on potentials like EAM. This approach dynamically couples and efficiently transfers cross-scale data while preserving atomic-level details of dislocations, stacking faults, and other defects. While efficient, the original localized formulation of QC could yield unphysical “ghost forces” under non-uniform deformation. Knapp et al. [77] subsequently developed the QC non-local method to eliminate these artifacts by non-localizing the sampling across the entire domain, achieving a consistent scale transition at an increased computational expense.

The CADD method [78,79] couples atomistic simulations with discrete dislocation plasticity (DDD) to study cross-scale dislocation behavior. In the CADD method, the core of data transfer between the atomistic and continuum scales lies in the two-way displacement coupling mechanism. The displacements of interface atoms in the atomistic region are directly mapped as displacement boundary conditions onto the finite element nodes of the continuum region, driving its deformation. Simultaneously, a layer of overlapping ghost atoms is introduced at the atomistic region boundary; their positions are generated by interpolating the continuum displacement field, acting as a “bridge” to seamlessly transmit macroscopic deformation to the real atoms. When a dislocation approaches the interface, the algorithm identifies the dislocation core via a detection band, eliminates the dislocation within the atomistic region, and relaxes the residual displacement, while simultaneously introducing an equivalent discrete dislocation into the continuum region. This achieves an automatic cross-scale transition of dislocation identity, thereby ensuring deformation compatibility and mass conservation.

The bridging scale method (BSM), introduced by Wagner [80] and Liu [81], couples MD and FE domains by scale-decomposing displacements and using the generalized Langevin equation (GLE) to damp fine-scale waves, preventing spurious reflections at the interface. Although primarily optimal for linear/weakly nonlinear regimes, its GLE framework requires expensive integral evaluations. To simplify this, Xiao and Belytschko [82] formulated the bridging domain method (BDM). The BDM replaces GLE with energy-based blending in an overlap region, achieving comparable wave suppression without complex integrals. Anciaux et al. [83] successfully simulated the rough surface contact behavior of copper using the bridging domain multiscale method.

### 3.2. Hierarchical Modeling

Hybrid multiscale modeling faces significant challenges in preventing unphysical artifacts (ghost forces and spurious wave reflections) at coupled domain interfaces. In contrast, hierarchical approaches avoid direct coupling artifacts by performing simulations sequentially: high-resolution models inform coarser ones through scale-bridging transfers. Consequently, robust information transfer, particularly from the molecular to continuum scale, becomes essential, as atomistic models capture defect-level phenomena invisible to continuum mechanics. Hierarchical methods commonly apply homogenization, especially the representative volume element (RVE) concept, to derive homogenized material properties. A cornerstone of this framework is the Cauchy–Born rule, valid for homogeneously deforming perfect crystals. It geometrically links macroscopic deformation to atomic motions, allowing interatomic potentials to directly determine the strain energy density for continuum constitutive models.

The hierarchical multiscale approach avoids coupling artifacts by transferring information across scales. Ghaffari [84] implemented this strategy for rolling contact fatigue, using molecular dynamics (MD) to simulate lubricant behavior and calculate the friction coefficient at the nanoscale. This parameter was transferred as a boundary condition to a macroscale finite element model (FEM) that analyzed contact stresses to predict fatigue life. Separately, Jiang et al. [85] employed MD to characterize the elastic/inelastic responses of materials under multiaxial loading. These results parameterized an isotropic elastic damage model implemented within the generalized interpolation material point (GIMP) method. While GIMP captured the essential nonlinear stress–strain behavior observed in MD, its continuum formulation produced unrealistically smooth damage surfaces—contrasting with MD’s atomistically rough fracture profiles—due to the simplified constitutive model.

### 3.3. Multiscale Modeling Based on Machine Learning

Multiscale material modeling, whether hybrid or hierarchical, demands substantial computing resources, with the computational cost growing prohibitively as the spatiotemporal scales increase. Machine learning (ML) [86,87] surrogates, however, offer transformative efficiency. Unlike traditional multiscale models, trained ML surrogates enable near-instantaneous predictions by mapping inputs to outputs without solving complex physics equations. Critical advantages include (1) decoupled costs, where the prediction time is independent of the simulation cost; (2) parallel deployment, where models support embarrassingly parallel inference; and (3) dimensionality reduction, capturing highly dimensional relationships that are inaccessible using conventional methods. For example, Jiahui Liu et al. [88] integrated the ZBL potential with the neuroevolution potential (NEP) framework to create a NEP-ZBL hybrid potential. They trained this potential for tungsten and validated it using properties including elastic constants, the melting point, and defect formation energies relevant to radiation damage. Employing this model in large scale molecular dynamics simulations involving 8.1 million atoms over 240 picoseconds, they systematically investigated the differences in primary radiation damage between bulk tungsten and tungsten thin films. Tatiana Kostiuchenko [89] addressed the challenge of chemical complexity in high-entropy alloys by developing low-rank potentials. These potentials efficiently map potential energy surfaces for Monte Carlo simulations, leveraging DFT-generated initial data. This approach significantly enhances the accuracy and computational efficiency of phase transition and short-range order predictions, revealing a previously unreported semi-ordered layered phase in the NbMoTaW alloy.

**Figure 6:**
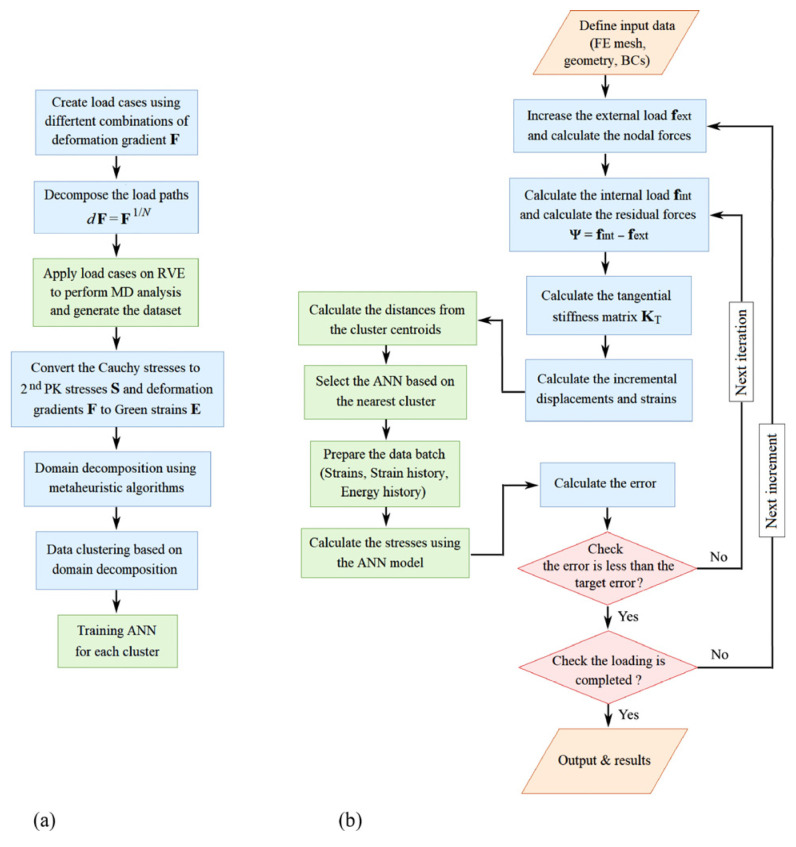
The flowchart of the proposed machine learning-based atomistic–continuum multiscale analysis: (**a**) the ML process on the atomic dataset and (**b**) the ML-based multiscale analysis. Reprinted with permission from Ref. [90]. Copyright 2023, Elsevier Ltd.

Khoei [90] developed a machine learning (ML)-based multiscale framework (Figure 6) to model nanocrystal plasticity. Molecular dynamics (MD) simulations analyzed nanoscale representative volume elements (RVEs) under diverse deformation paths, generating training data for ML. A parametric study determined RVE sizes, ensuring scale separation for homogenized mechanical properties. The framework uses a multilayer perceptron artificial neural network (MLP-ANN) as a surrogate to map deformation gradients to stress responses, bypassing explicit homogenization. Validation confirmed high accuracy in predicting RVE responses under arbitrary deformations.

Hierarchical multiscale modeling faces significant information transfer challenges between scales. Xiao [91] addressed this through a physics-aware ML pipeline: Molecular dynamics simulations first generate nanoscale datasets, training simultaneous defect classifiers and stress regressors. These ML models subsequently inform continuum simulations of macroscopic mechanical behavior. Complementing this, Khoei [92] developed a partitioned approach for Ni-based superalloys. Here, MD-derived stress–strain data undergo K-means clustering by strain magnitude, enabling specialized feedforward neural networks (FFNNs) for each deformation regime. Bayesian regularization optimizes FFNN parameters to prevent overfitting. Crucially, each FEM Gauss point employs a dedicated FFNN to output local stress tensors and tangent moduli (∂σ/∂ε), bypassing real-time atomistic simulations. Both frameworks demonstrate how ML-driven scale-bridging enhances computational efficiency while preserving microstructural sensitivity.

Despite demonstrating significant computational acceleration capabilities in multiscale materials simulation, machine learning (ML) still faces two critical challenges: overfitting risks and a lack of physical interpretability. On the one hand, the performance of ML models heavily relies on the quality and coverage of training data. In materials science, acquiring high-precision simulation data is prohibitively expensive, resulting in limited dataset sizes. When the model complexity is excessive or the data representation is insufficient, overfitting becomes highly probable. For instance, neural networks trained on small datasets for predicting coating wear rates may amplify data noise, leading to substantial prediction deviations [93]. On the other hand, widely adopted black-box models struggle to establish explicit connections to physical mechanisms, which fundamentally constrains the depth of material design. Coating material development inherently requires understanding the causal chain in “structure–property” relationships, yet ML can only provide statistical mappings between inputs and outputs. Taking corrosion potential prediction as an example [94], while an ML model might deliver predictions with high accuracy, it fails to reveal quantitative relationships between key physical quantities, such as the electron work function and interfacial adsorption energy. This absence of physical mechanisms severely impedes the progress of mechanism-driven material optimization. To overcome these inherent limitations of ML in material simulation, research frontiers are advancing paradigm shifts through multi-dimensional integration strategies. The core pathway involves the deep embedding of physical constraints, exemplified by the development of physics-informed neural networks (PINNs) [95]. Such approaches integrate fundamental principles, including energy conservation and constitutive equations as soft constraints into the loss function, compelling models to adhere to physical laws even in data-sparse regions. This evolution marks a transition from purely data-driven ML toward a new paradigm of physics-coupled machine learning.

## 4. Prospect

Multiscale simulations of nanocoatings begin with atomic-scale interface modeling between coatings and substrates. Density functional theory (DFT) calculates interfacial binding energies to evaluate thermodynamic stability, including coating surface relaxation effects induced by substrate bonding. For stable configurations identified through this analysis, molecular dynamics (MD) simulations probe tribological behavior: sliding contact simulations determine friction coefficients, the atomic virial theorem quantifies surface stress distributions, non-equilibrium MD characterizes interfacial heat diffusion, and strain fluctuation methods compute the elastic moduli of coating–substrate systems. This hierarchical workflow bridges quantum-scale adhesion to macroscopic wear properties.

Gan et al. [96] bridged the atomic-to-macroscopic scales for coating thermal stress prediction: First-principles calculations determined rare-earth tantalate thermomechanical properties (thermal expansion via QHA, conductivity via the Debye model, and elasticity via stress–strain relations). These properties informed coupled thermo-mechanical FEM simulations to quantify stress fields. A machine learning surrogate model that was trained on FEM datasets identified electronegativity, a low Poisson’s ratio, and minimized heat capacity/thermal expansion as dominant low-stress indicators. Crucially, validation on novel compositions YTaO_4_ and (Ho_0.5_Er_0.5_)TaO_4_ demonstrated that the RF model generalizes beyond its training data, enabling the rapid screening of thermal barrier coatings.

Future work will develop ML models (e.g., ANNs and CNNs) to predict coating wear resistance through multiscale data integration. Feature vectors encoding atomic composition and microstructure descriptors will serve as inputs, while macroscale properties such as the elastic modulus, friction coefficient, and surface tension will constitute model outputs. Iterative active learning will train these models: (1) initial predictions will guide the experimental synthesis of novel coatings, (2) experimental measurements will validate and expand the training dataset, and (3) Bayesian optimization will update the model. This closed-loop framework will ultimately establish composition–structure design rules for wear-resistant nanocoatings, validated through uncertainty-quantified predictions and targeted experimentation.

Building an efficient closed-loop validation system integrating multiscale simulation and experimental characterization is a core challenge urgently requiring a solution in future research. Sole reliance on simulation results to guide coating design and performance prediction carries significant risks. An efficient closed-loop validation system must meet several key requirements. First, it necessitates the development of in situ and high-throughput experimental characterization techniques to precisely capture critical microstructural evolution and macroscopic performance responses during coating fabrication and service, generating quantitative data matching the spatiotemporal scales of simulations. Second, an automated, standardized data interface and assimilation platform must be established to enable real-time, seamless feedback of experimental observation data into corresponding scale simulation modules, thereby dynamically calibrating model parameters, refining constitutive relationships, and validating cross-scale correlation hypotheses. Ultimately, based on the validated high-confidence models, an inverse design is employed to propose novel coating compositions, structures, or processing schemes, whose performance is then verified experimentally. Only by efficiently operating the closed loop of model prediction, experimental validation, model optimization, and design iteration can the entire chain be genuinely bridged, from atomic/molecular-scale understanding to macroscopic performance control. This will significantly enhance the precision, efficiency, and success rate of coating material development, ultimately driving multiscale simulation toward a paradigm shift, from explaining phenomena to precise design.

## 5. Conclusions

Multiscale simulation of nanowear-resistant coatings has emerged as a critical research frontier in materials science, driven by applications demanding extreme durability (e.g., turbine engines and biomedical implants). This review synthesized current methodologies bridging the atomic-to-macroscales to address fundamental challenges: atomic adhesion governs interface stability, nanoscale grain dynamics affect toughness, and microscale topology determines friction behavior. We systematically analyzed how density functional theory, molecular dynamics, coarse-grained techniques, and continuum mechanics collectively decode structure–property relationships across scales, enabling the predictive design of next-generation wear-resistant coatings.

Atomic-scale simulations decode nanocoating wear fundamentals: DFT calculates interfacial adhesion energies and defect formation barriers, while MD replicates wear processes through controlled sliding tests. By correlating atomic events (e.g., bond rupture sequences and dislocation-mediated plasticity) with macroscopic material loss, these methods establish causal structure–performance relationships essential for predictive coating design.

While molecular dynamics simulations capture atomistic wear mechanisms, their applicability is restricted to nanometer-scale volumes and nanosecond timescales. Macroscale phenomena—including fatigue damage evolution and stress distribution in engineering components—require finite element modeling. Bridging these scales through concurrent coupling methods (e.g., domain decomposition) or hierarchical parameter passing enables multiscale frameworks that retain atomistic fidelity while accessing engineering-relevant dimensions. Such integration provides predictive capabilities for coating design, validated through coordinated experimentation (e.g., TEM characterization of simulated dislocation structures). Future advancements demand physics-informed machine learning surrogates to accelerate the exploration of coating design spaces, ultimately enabling the on-demand development of nanocoatings for aerospace, automotive, and biomedical applications.

## Figures and Tables

**Table 1:** Comparison of simulation methods for nanowear-resistant coatings.

Method	Advantages	Disadvantages	Applicable Scenarios	Experimental Validation Methods
Density functional theory (DFT)	1. High accuracy 2. Calculates electronic structure, interfacial adhesion, and defect formation energy 3. No empirical parameters required	1. Extremely high computational cost 2. Limited to small systems 3. Time scale restricted to picoseconds	1. Coating/substrate interface adhesion energy 2. Corrosion mechanisms 3. Intrinsic mechanical properties	1. XPS/AES [65] 2. XRD [66] 3. STEM (atomic interface structure) [67]
Molecular dynamics (MD)	1. Atomic resolution 2. Simulates dynamic processes	1. Limited time scale (nanoseconds) 2. Limited spatial scale (sub-micron) 3. Accuracy depends on force field reliability	1. Nanoscale wear mechanisms 2. Coating plastic deformation and crack propagation	1. In situ TEM nanoscratch [68] 2. Nanoindentation [69] 3. AFM/STM (surface evolution) [70] 4. SEM/TEM (coating morphology) [70]
Monte Carlo (MC)	1. High computational efficiency 2. Suitable for stochastic processes 3. No iteration convergence issues	1. No dynamic evolution information 2. Depends on probabilistic model accuracy 3. Difficult for non-equilibrium processes	1. Coating deposition 2. Atomic diffusion and grain boundary evolution	1. SEM/TEM (coating morphology) [71] 2. UV–vis [72]
Finite element method (FEM)	1. Handles complex macroscopic geometries 2. Efficient stress/temperature field computation 3. Supports multiphysics coupling	1. Cannot describe atomistic mechanisms 2. Requires empirical constitutive models 3. Poor accuracy at nanoscale	1. Macroscopic stress distribution in coatings 2. Laser cladding process optimization	1. Strain gauges/DIC [73] 2. Thermocouple/IR imaging [74]

## Data Availability

No new data were created or analyzed in this study. Data sharing is not applicable to this article.
